# Benzothiazole Treatment Regulates the Reactive Oxygen Species Metabolism and Phenylpropanoid Pathway of *Rosa roxburghii* Fruit to Delay Senescence During Low Temperature Storage

**DOI:** 10.3389/fpls.2021.753261

**Published:** 2021-10-25

**Authors:** Boyu Dong, Hongmin Tang, Dequan Zhu, Qiuping Yao, Hongqiang Han, Kequn He, Xiaochun Ding

**Affiliations:** ^1^College of Ethnic-Minority Medicine, Guizhou Minzu University, Guiyang, China; ^2^State Key Laboratory of Plant Resources Conservation and Sustainable Utilization, South China Botanical Garden, Chinese Academy of Sciences, Guangzhou, China

**Keywords:** *Rosa roxburghii*, benzothiazole, fruit quality, reactive oxygen species metabolism, phenylpropanoid pathway

## Abstract

*Rosa roxburghii* fruit were used as research objects to study the effects of different concentrations of benzothiazole (BTH) treatment on quality parameters, reactive oxygen species (ROS) metabolism, and the phenylpropanoid pathway during storage at 4°C for 14days. Results showed that BTH effectively delayed senescence with lower decay incidence, weight loss, and lipid peroxidation level and maintained the quality with higher contents of total soluble solid (TSS) content, titratable acidity (TA) in *R. roxburghii* fruit. Moreover, BTH increased hydrogen peroxide (H_2_O_2_) content, superoxide anion (O_2_^•−^) production rate, and the activities and expression of superoxide dismutase (SOD), catalase (CAT), ascorbate peroxidase (APX), glutathione (GSH) reductase (GR), monodehydroascorbate reductase (MDHAR), dehydroascorbate reductase (DHAR), and peroxidase (POD), and the contents of GSH and ascorbic acid (AsA), but reduced the oxidized GSH (GSSG) content. In addition, the activities and gene expression of phenylalanine ammonia lyase (PAL), cinnamate 4-hydroxylase (C4H), and 4-coumarate-CoA ligase (4CL) and the concentrations of flavonoids, total phenols, and lignin were significantly elevated by BTH. These findings imply that BTH can delay senescence and maintain the quality of *R. roxburghii* fruit by modulating ROS metabolism and the phenylpropanoid pathway under low-temperature conditions.

## Introduction

*Rosa roxburghii* (*R. roxburghii*), a perennial deciduous shrub from the Rosaceae family, is widely distributed in southwest China. The vitamin C and superoxide dismutase (SOD) contents of *R. roxburghii* fruit are particularly rich and exhibit considerable promotion prospects in the market (Liu et al., 2016; Yang et al., 2020). However, *R. roxburghii* is prone to rot due to water loss, softening, pathogen infection, and cell wall degradation during storage at room temperature. Rotting seriously affects the appearance and quality of *R. roxburghii* fruit, reducing their commercial value. *R. roxburghii* fruit can only be stored for approximately 8days at room temperature. At present, studies on the postharvest storage of *R. roxburghii* fruit, including those regarding 1-methylcyclopropene and bagging treatments, are relatively rare (Fan et al., 2019; Zhang et al., 2020). To date, reports on the effects of inducer treatment on the phenylpropanoid pathway in *R. roxburghii* fruit and reactive oxygen species (ROS) metabolism remain few.

The structural analogue of salicylic acid, benzothiazole (BTH) has confirmed to induce the production and improvement of disease resistance in various plants (Lavergne et al., 2017). BTH can effectively increase the postharvest quality and delay the senescence of pear (Li et al., 2020a), muskmelon (Liu et al., 2014; Ge et al., 2015), grape (Ruiz-García et al., 2013), and strawberry (Cao et al., 2011) fruit. Previous studies have demonstrated that BTH treatment can induce disease resistance and maintain fruit quality; these activities are closely related to ROS metabolism (Ge et al., 2015) and the phenylpropanoid pathway (Liu et al., 2014).

Reactive oxygen species have been confirmed to act directly on pathogens and function as a defensive gene signals in plant defense responses (Oliveira et al., 2016). However, excessive ROS will damage cells and destroy the integrity of the cell membrane, leading to the accelerated senescence of fruit (Ray et al., 2012; Nath et al., 2017). Plants may eliminate excess ROS through the ascorbic acid (AsA)-glutathione (GSH) cycle, which is critical for balancing ROS metabolism and increasing the antioxidant capacity of fruit and vegetables (de Freitas-Silva et al., 2017; Zhu et al., 2019). Rasool et al. (2013) suggested that the AsA–GSH cycle comprises antioxidant enzymes and compounds, including SOD, catalase (CAT), ascorbate peroxidase (APX), GSH reductase (GR), monodehydroascorbate reductase (MDHAR), dehydroascorbate reductase (DHAR), AsA, and GSH. Research has demonstrated that increasing the activity and gene expression of enzymes involved in ROS metabolism increases the antioxidant capacity of blueberry (Ge et al., 2019b) and pear (Li et al., 2020a) fruit after harvest.

The phenylpropanoid pathway is a unique secondary metabolism in plants, and it is important in the development of plant disease resistance. Many major antibacterial substances, such as lignin, flavonoids, and phenolic compounds are directly or indirectly generated by the phenylpropanoid pathway (Ge et al., 2012). The buildup of phenylpropanoid pathway products can boost the antioxidant potential of muskmelons and blueberries (Liu et al., 2014; Ge et al., 2015, 2019b). The effects of BTH treatment on the antioxidant capacity and phenylpropanoid pathway of *R. roxburghii* remain unclear.

The current study aimed to investigate the effects of BTH treatment on the following: (1) the quality including: weight loss rate, decay incidence, malondialdehyde (MDA), cell membrane permeability, titratable acidity (TA), soluble solids (TSS), and AsA content of *R. roxburghii* fruit during low-temperature storage, (2) the ROS metabolism of *R. roxburghii* fruit during low-temperature storage, and (3) the phenylpropanoid pathway of *R. roxburghii* fruit. That is, this research intended to evaluate the potential of BTH in enhancing the postharvest quality and delaying the senescence of *R. roxburghii* fruit under low-temperature conditions.

## Materials and Methods

### Fruit and Chemicals

*R. roxburghii* fruit harvested at 85days after full blossom (about 70% maturity) without mechanical damage were obtained from an orchard planted with the AsA–GSH cycle comprises *R. roxburghii* in Anshun, Guizhou Province, China. Sigma-Aldrich provided support for BTH.

### Treatment

Undamaged fruit were selected and washed three times with distilled water. All the selected fruit were immersed in 25, 50, 100, and 200mgL^−1^ of BTH dissolving with 0.1mlL^−1^ Tween-80 for 10min, with distilled water as the control. All the processed fruit were air-dried at ambient temperature (22°C) before being stored at 4°C for 14days. Each treatment consisted of three replicates, and each replicate comprised 80 fruits.

### Sample Collection

On days 0, 2, 4, 6, 8, 10, 12, and 14 after BTH treatment, tissues were detached from 3 to 6mm below the skin and around the equator of a fruit. Consequently, the collected samples were frozen in liquid nitrogen and immediately stored at −80°C.

### Determination of Weight Loss and AsA Content

The weighing method used for evaluation and measurement was based on the following formula: weight loss rate/%=[(quality before storage−quality after storage)/quality before storage]×100. The AsA content was assayed by applying the method of Ge et al. (2015) and was expressed as g kg^−1^ fresh weight (FW). The optimal concentration of BTH was determined on the basis of the weight loss rate and AsA content.

### Determination of Decay Incidence

Decay index is divided into six levels. Level 0: no decay and mold; Level 1: small brown spots, no obvious decay area; Level 2: decay area <1/4 of the fruit surface area; Level 3: 1/4<decay area<1/3 of the fruit surface area; Level 4: 1/3<decay area<1/2 of the fruit surface area; and Level 5: decay area>1/2 of the fruit surface area. The decay incidence (%) was calculated according to the following equation: ∑ (number of fruits×decay index)/(total number of fruits×the highest level of decay index)×100.

### Determination of TSS, TA, Integrity of the Cell Membrane, and MDA Content

Pulp tissues (2.0g) from 10 fruits were homogenized. The supernatant was dropped onto a TF-65 handheld refractometer. Total soluble solid (TSS) content was expressed in %. Then, acid–base titration was performed with 0.1molL^−1^ of sodium hydroxide standard solution. The method of Sayyari et al. (2009) was adopted as a reference for cell membrane integrity. A sample was cut into 10 slices with a thickness of 2mm. After soaking in double distilled water at 25°C for 3h, conductivity was measured using a conductivity meter and denoted as CI. Conductivity was measured again after 30min of boiling. After cooling to room temperature (22°C), the final conductivity was denoted as *CF*. Cell membrane integrity was estimated using the following formula: cell membrane integrity (%)=[1−(CI/*CF*)]×100. The study of Gao et al. (2018) was used as a reference for determining MDA content. Absorbance was measured at 450, 532, and 600nm. MDA content was expressed in mmol kg^−1^ FW.

### Measurement of the Hydrogen Peroxide (H_2_O_2_) Content, Superoxide Anion (O_2_^•−^) Production Rate, GSH, and Oxidized GSH (GSSG) Content

The method of Ge et al. (2015) was used as a reference for determining the production rate of O_2_^•−^. The rate of O_2_^•−^ production was measured using an mmol g^−1^ min^−1^ FW scale. H_2_O_2_ content was determined in accordance with the method of Ren et al. (2012). Subsequently, 3g of frozen tissue was homogenized in 3ml of cold acetone at 4°C and then centrifuged at 9,000×*g* for 20min at 4°C. Thereafter, 1ml of supernatant with 200μl of 20% titanium tetrachloride dissolved in concentrated hydrochloric acid (HCl, v/v) was used in the reaction. The titanium–hydroperoxide complex precipitate was filtered three times in cold acetone before being dissolved in 3ml of 1M sulfuric acid and centrifuged for 10min at 4°C at 9,000×*g*. H_2_O_2_ content was measured using an absorbance of 410nm and expressed in μmol g^−1^ FW. GSH content was assayed by applying the method of Ge et al. (2015) and expressed as μmol g^−1^ FW. GSSG contents were determined using GSSG Detection Assay Kit (Beyotine, Shanghai, China) and were expressed as μmol g^−1^ FW.

### Extraction of Crude Enzyme and Enzymatic Activity Assays

All the extractions were performed at 4°C. Liquid nitrogen was used to grind frozen tissues (1.0g) into powder, which was subsequently extracted with the following solutions:

SOD and CAT: 3.0ml of phosphate buffer (pH 7.4, 0.05M) with 5.0mM of dithiothreitol (DTT) and 10gL^−1^ of polyvinyl polypyrrolidone (PVPP). SOD activity was determined using the method described by Ren et al. (2012). The reaction mixture was composed of 0.4ml of crude enzyme, 1.5ml of 50mM sodium phosphate buffer (pH 7.8), 0.3ml of 130mM methionine, 0.3ml of 100μM ethylenediaminetetraacetic acid (EDTA), 0.3ml of 750μM nitro blue tetrazolium (NBT), and 0.3ml of 20μM riboflavin. Absorbance at 560nm was used to track the development of blue formazan. Activity was denoted by U·g^−1^ protein, where U=50% inhibition of NBT. CAT activity was determined using the method described by Ren et al. (2012). First, 3ml of 0.01molL^−1^ H_2_O_2_ and 0.3ml of crude enzyme extract were used in the reaction. The absorbance of the supernatant was then measured at 240nm.

APX and DHAR: 3.0ml of phosphate buffer (pH 7.5, 0.1M) with 1.0mM of EDTA. APX activity was determined as described by Ren et al. (2012). Briefly, 2ml of 100mM sodium phosphate buffer (pH 7.5), 0.5ml of 30% (v/v) H_2_O_2_, and 0.2ml of enzyme extract were used in the assay mixture. APX activity was measured in units of U mg^−1^ protein, i.e., 1U=0.01 Δ340 min^−1^. DHAR activities were measured using the method of Ren et al. (2012) and expressed as U mg^−1^ protein, where 1U=0.01A290 min^−1^.

GSH reductase: 0.1M of phosphate buffer (pH 7.5, 1mM of EDTA). The GR-dependent oxidation of NADPH was used to determine GR activity at 340nm by using the method of Ren et al. (2012). An enzyme reaction mixture that contained 0.2ml of crude enzyme, 3ml of 100mM sodium phosphate buffer (pH 7.5), 30μl of 3mM NADPH, and 0.1ml of 10mM oxidized GR was used. Activity was measured in units of U mg^−1^ protein.

MDHAR: 0.04M of phosphate buffer (pH 7.5, 20gL^−1^ of PVPP, and 5mM of β-mercaptoethanol). MDHAR activities were measured using the method of Ren et al. (2012). Activities were measured in units of U mg^−1^ protein, where 1U=0.01A340 min^−1^.

Peroxidase (POD): 0.05M of phosphate buffer (pH 7.5, 1ml of L^−1^ Triton X-100, and 20gL^−1^ of PVPP). POD activity was assayed in accordance with the method of Liu et al. (2014). The oxidation of guaiacol into tetraguaiacol was observed spectrophotometrically at 470nm for 2min. Activity was expressed as U mg^−1^ protein, where 1U=0.01 Δ470 min^−1^.

Phenylalanine ammonia lyase (PAL): 5ml of sodium borate buffer (0.05M, pH 8.8, 5mM of mercaptoethanol, 2mM of EDTA, and 20gL^−1^ of PVPP). PAL activity was determined using the method of Liu et al. (2014). First, 1ml of crude enzyme was incubated with 3ml of l-phenylalanine for 60min at 37°C. PAL activity was evaluated by measuring absorbance at 290nm. Activity was indicated in units of U·mg^−1^ protein. Here, U=0.01 ΔOD290 min^−1^.

Cinnamate 4-hydroxylase (C4H): 5ml of 50mM Tris–HCl buffer (pH 8.9) with 4mM of MgCl_2_, 15mM of mercaptoethanol, 10M of leupeptin, 5mM of l-ASA, 1mM of phenylmethylsulfonyl fluoride, 1g/L (w/v) of PVPP, and 10% (v/v) glycerin. C4H activity was determined in accordance with the method of Liu et al. (2014). It was measured in 0.01 ΔOD340/mg protein.

4-Coumarate-CoA ligase (4CL): 5ml of 0.2M Tris–HCl buffer (pH 7.5) with 25% glycerin and 0.1M of DTT. The activity of 4CL was assessed using the technique of Liu et al. (2014). Here, 0.5ml of crude enzyme was added to a reaction mixture that contained 0.45ml of 75mM MgCl_2_, 0.15ml of 1M coenzyme A (CoA), 0.15ml of 0.8mM adenosine triphosphate, and 0.15ml of 2mM p-cumaric acid. Activity was measured in U·mg^−1^ protein by using U=0.01 ΔOD333 min^−1^.

### Analysis of the Metabolite Content in the Phenylpropanoid Pathway

Total phenolic, flavonoid, and lignin contents were extracted and measured using the method described by Liu et al. (2014). Total phenolic and lignin concentrations were measured at OD280 and OD325, respectively and determined as mg·Kg^−1^ FW.

### RNA Extraction and First-Strand Complementary DNA Synthesis

RNA was extracted from tissues (1.0g) by using an RNeasy Plant Mini Kit (Takara, Japan) following meticulous procedures. First-strand complementary DNA (cDNA) was synthesized from 1μg of total RNA by utilizing a Reverse-iT™ 1st Strand Synthesis Kit (Takara, Japan) and strictly following the manufacturer’s instructions.

### Real-Time Quantitative RT-PCR

A real-time quantitative PCR experiment was conducted by adding 10μl of SYBR™ Green qPCR Master Mix, 1μl of template cDNA, and 1μl of 10μM forward and reverse primers to each well with a 20μl reaction volume. An ABI PRISM™ 7,000 Sequence Detection System was used for measurements (Applied Biosystems). Cycling conditions were 95°C for 10min, followed by 40cycles of 15s at 95°C and 40s at 60°C. The 2^−ΔΔt^ formula was used to calculate the target gene expression and the *actin* gene was used as reference. Three duplicates of real-time PCR were conducted for each sample. Primers were indicated in Supplementary Table 1.

### Data Analysis

By using SPSS 19.0, all data on enzyme activities and substance contents were evaluated using one-way ANOVA of least significant difference (*p*<0.05).

## Results

### Effects of Different BTH Concentrations on Weight Loss, Decay Incidence, and AsA Content

**Figure 1:**
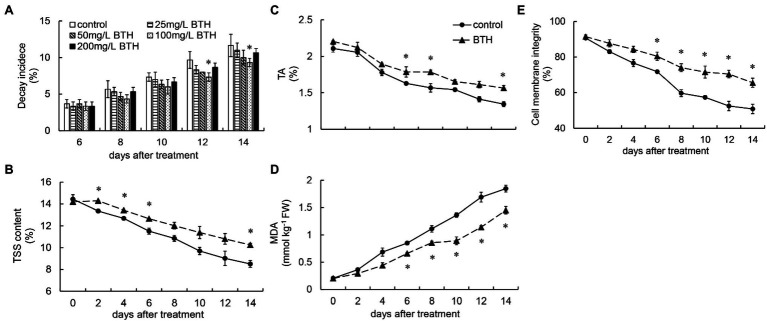
Effect of benzothiazole (BTH) treatment on the decay incidence **(A)**, titratable acidity (TA; **B**), total soluble solid (TSS; **C**), malondialdehyde (MDA) content **(D)**, and cell membrane integrity **(E)** of *R. roxburghii* fruit. The significant differences at a level of 0.05 by Fisher’s least significant difference (LSD) between the control and BTH-treated fruit are indicated by asterisks (*). Vertical bars represent the standard errors of the means (±SE).

The weight loss of the BTH-treated fruit at 25, 50, 100, and 200mg/L was compared with that of the control fruit, as presented in Table 1. As shown in the table, 100mg/L of BTH significantly slowed down weight loss during the storage period. As shown in Figure 1A, from the sixth day of storage, the fruits of the different concentrations of BTH treatment and the control appeared to decay. On days 12 and 14, the fruit decay rate of the 100mg/L BTH treatment was significantly lower than that of the control (Figure 1A). Table 2 provides the AsA content of the BTH-treated and control fruit during the storage period. In this table, 100mg/L of BTH exerted the best effect on increasing AsA content compared with the 25, 50, and 200mg/L of BTH treatments of *R. roxburghii* fruit. Therefore, the concentration of 100mg/L of BTH was selected for the subsequent experiments.

**Table 1:** Effects of BTH treatment at different concentrations on the weight loss in *R. roxburghii* fruit.

Treatment	2Days	4 Days	6 Days	8 Days	10 Days	12 Days	14 Days
Control	3.58±0.07^a^	5.29±0.10^a^	10.91±0.69 ^a^	11.80±0.63^a^	14.45±0.34^a^	16.40±0.73^a^	17.43±0.34^a,b^
BTH (mg/L)							
25	3.30±0.35^a^	5.13±0.46^a,b^	10.57±0.24^a^	11.22±0.51^a,b^	14.83±0.69^a^	15.52±0.78^a,b^	17.86±0.44^a^
50	3.12±0.17^a^	4.86±0.33^a,b^	9.23±0.17^b^	10.18±0.47^b,c^	13.57±0.92^a,b^	15.77±0.79^a^	16.44±0.38^b,c^
100	3.01±0.03^a^	4.43±0.43^b^	7.80±0.81^c^	9.70±0.46^c^	12.34±1.82^b^	14.41±0.33^b^	16.23±0.83^c^
200	3.57±0.64^a^	5.06±0.42^a,b^	8.74±0.21^b^	10.53±0.81^b,c^	14.76±0.33^a^	15.85±0.66^a^	17.97±0.70^a^

Different letters indicate significant differences between the BTH-treated and control fruit (*p*<0.05).

**Table 2:** Effects of BTH treatment at different concentrations on ascorbic acid (AsA) content in *R. roxburghii* fruit.

Treatment	0Day	2 Days	4 Days	6 Days	8 Days	10 Days	12 Days	14 Days
Control	7.39±0.35^a^	7.53±0.13^a^	7.49±0.10^a^	7.48±0.15^a^	7.99±0.37^a^	7.58±0.25^a^	7.79±0.46^a,b^	6.41±0.22^a^
BTH (mg/L)								
25	7.52±0.27^a^	7.65±0.81^a^	7.83±0.55^a,b^	8.23±0.54^a^	8.83±0.25^a,b^	8.14±0.60^a^	7.34±0.74^a^	7.54±0.57^b,c^
50	7.45±0.19^a^	7.63±0.52^a^	7.52±0.59^a^	8.11±0.20^a^	9.43±0.45^b^	8.43±0.39^a^	8.65±0.23^b,c^	6.76±0.57^a,b^
100	7.43±0.36^a^	8.21±0.43^a^	8.82±0.27^b^	9.22±0.52^b^	11.71±1.02^c^	11.02±0.84^b^	9.48±0.32^c^	8.25±0.67^c^
200	7.24±0.37^a^	7.78±0.30^a^	8.23±1.00^a,b^	9.65±0.69^b^	9.75±0.61^b^	10.12±0.91^b^	8.73±0.61^b,c^	7.32±0.77^a,b,c^

Different letters indicate significant differences between the BTH-treated and control fruit (*p*<0.05).

### Effects of BTH Treatment on TSS, TA, MDA Content, and Cell Membrane Integrity

Throughout the storage period, TA continued to decrease in the control and BTH-treated fruit. However, BTH treatment slowed the decline of TA, and the BTH-treated fruit exhibited considerably higher TA levels than the control fruit on days 6–8 and 14 (Figure 1B). Similar to TA, TSS content continued to decrease in the control and BTH-treated fruit. However, TSS content in the BTH-treated fruit was always higher than that in the control fruit. It was considerably higher on days 2–6 and 14 (Figure 1C). The MDA content of the BTH-treated and control fruit increased throughout the storage period. However, BTH remarkably inhibited MDA increase on days 6–14 (Figure 1D). The BTH-treated fruit performed substantially better than the control fruit, with cell membrane integrity being considerably higher than that of the control group on days 6–14 (Figure 1E).

### Changes in O_2_^•−^ Production Rate, H_2_O_2_ Content, and SOD, CAT, and POD Activities After BTH Treatment

**Figure 2:**
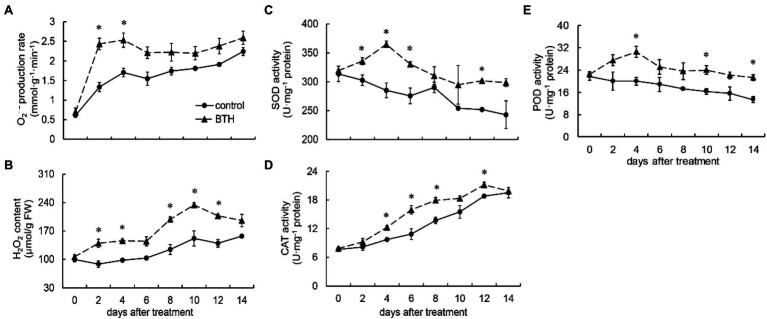
Changes in superoxide anion (O_2_^•−^) production rate **(A)**, hydrogen peroxide (H_2_O_2_) content **(B)**, and the activities of superoxide dismutase (SOD; **C**), catalase (CAT; **D**), and peroxidase (POD; **E**) in *R. roxburghii* fruit after BTH treatment during low temperature storage. The significant differences at a level of 0.05 by LSD between the control and BTH-treated fruit are indicated by asterisks (*). Vertical bars represent the standard errors of the means (±SE).

After BTH treatment, O_2_^•−^ production rate increased rapidly on days 0–4 and reached the highest value on day 4, which was 48.2% higher than that of the control. During storage, the O_2_^•−^ production rate of the BTH-treated fruit was higher than that of the control fruit. Moreover, it was considerably higher on days 2–4 (Figure 2A). H_2_O_2_ content increased on days 0–10 in the BTH-treated fruit and reached its peak on day 10, which was 54.3% higher than that of the control fruit, and then decreased. The H_2_O_2_ concentration of the BTH-treated fruit was greater than that of the control fruit throughout the preservation period. It was considerably higher on days 2–4 and 8–12 (Figure 2B). The SOD activity of the BTH-treated fruit was greater than that of the control fruit during storage, and it peaked on day 4, being 27.8% greater than that of the control fruit (Figure 2C). Substantial differences existed between the control and BTH-treated fruit on days 2–6 and 12. CAT activity increased during the storage period in two groups. On days 4–8 and 12, the CAT activity of the BTH-treated fruit was considerably higher (Figure 2D). On days 0–4, the POD activity of the BTH-treated fruit increased and peaked on day 4, which was 52.6% higher than that of the control fruit, before declining (Figure 2E). On days 4, 10, and 14, the POD activity of the BTH-treated fruit was considerably higher than that of the control group. In addition, the POD activity of the control fruit was always reduced during storage.

### Effects of BTH Treatment on APX, GR, MDHAR, and DHAR Activities and GSH, GSSG Content

**Figure 3:**
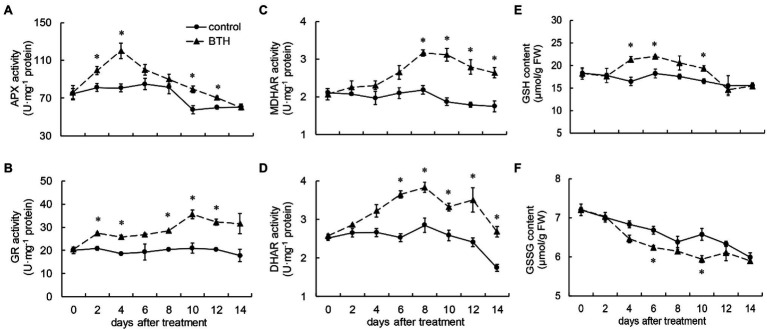
Changes in the activities of ascorbate peroxidase (APX; **A**), Glutathione reductase (GR; **B**), monodehydroascorbate reductase (MDHAR; **C**), and dehydroascorbate reductase (DHAR; **D**) and the content of glutathione (GSH; **E**) and GSSG **(F)** in *R. roxburghii* fruit after BTH treatment during low temperature storage. The significant differences at a level of 0.05 by LSD between the control and BTH-treated fruit are indicated by asterisks (*). Vertical bars represent the standard errors of the means (±SE).

The APX activity of the fruit increased rapidly after BTH treatment on days 0–4. It peaked on day 4, when it was 48.9% greater than that of the control fruit, and then declined. During storage, the BTH-treated fruit exhibited a more activated APX than the control fruit, and significant differences were observed between the control and BTH-treated fruit on days 2, 4, 10, and 12 (Figure 3A). BTH treatment boosted GR activity, which increased rapidly after 8days and peaked on day 10, when it was 39.6% greater than that of the control fruit (Figure 3B). MDHAR activity in the BTH-treated fruit increased on days 0–8, peaked on day 8 (45.1% greater than that of the control group), and then declined on days 8–14. On these days, the MDHAR activity of the BTH-treated fruit was substantially higher than that of the control fruit (Figure 3C). DHAR activity increased from days 0 to 8 and peaked on day 8 in the BTH-treated fruit, 34.4% higher than that of the control fruit (Figure 3D). As shown in Figure 3E, the GSH content peaked on day 6, after BTH treatment, when it was 20.8% higher than that of the control fruit. The BTH-treated fruit had considerably higher GSH than the control fruit on days 4–6 and 10 (Figure 3E). However, BTH-treated fruit showed lower GSSG levels than the control fruit throughout the storage period (Figure 3F).

### Effects of BTH Treatment on PAL, C4H, and 4CL Activities and Total Phenolic, Flavonoid, and Lignin Contents

**Figure 4:**
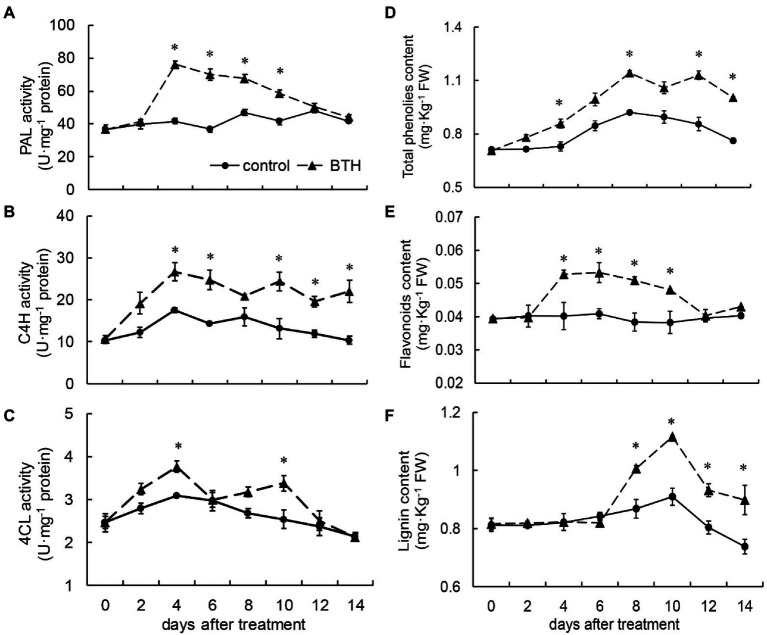
Changes in the activities of phenylalanine ammonia lyase (PAL; **A**), cinnamate 4-hydroxylase (C4H; **B**), 4-coumarate-CoA ligase (4CL; **C**), and the content of total phenolic **(D)**, flavonoids **(E)**, and lignin **(F)** in *R. roxburghii* fruit after BTH treatment during low temperature storage. The significant differences at a level of 0.05 by LSD between the control and BTH-treated fruit are indicated by asterisks (*). Vertical bars represent the standard errors of the means (±SE).

PAL activity was consistently higher in the BTH-treated fruit than in the control fruit throughout the storage period. On day 4, the PAL activity of the BTH-treated fruit peaked at 83.2% higher than that of the control fruit. A significant difference was observed between the two groups of fruit on days 4–10 (Figure 4A). C4H activity increased on days 0–4. The first peak, which appeared on day 4, was 51.8% higher than that of the control fruit and then declined. The second peak appeared on day 10 (85.0% higher than that in the control group). The BTH-treated fruit exhibited considerably higher C4H activity than the control fruit on days 4–6 and 10–14 (Figure 4B). After BTH treatment, two peaks of 4CL activity appeared in *R. roxburghii* fruit on days 4 and 10. They were 21.9 and 33.3% greater than that of the control fruit, as shown in Figure 4C. BTH increased total phenolic content, which peaked on day 8 and was 24.0% greater than that of the control fruit. On days 4, 8, 12, and 14, the total phenolic content of the BTH-treated fruit was considerably greater than that of the control fruit (Figure 4D). The total phenolic content of the BTH-treated fruit was always higher than that of the control fruit during preservation. BTH increased flavonoid content, reaching a peak on day 6, which was 30.3% higher than that of the control fruit. The BTH-treated fruit contained considerably higher amounts of flavonoids on days 4–10, while the flavonoid content of the control fruit remained at a low level throughout the storage period (Figure 4E). The lignin contents of the BTH-treated and control fruit remained basically consistent during the first 6days, while the lignin content of the BTH-treated fruit increased from day 8 and reached a peak on day 10 (22.6% higher than that of the control fruit). On days 8–14, BTH considerably increased lignin content (Figure 4F).

### Effects of BTH Treatment on the Expression of *RrSOD*, *RrCAT*, *RrPOD*, *RrAPX*, *RrGR*, *RrMDHAR*, *RrDHAR*, *RrPAL*, *RrC4H*, and *Rr4CL*

**Figure 5:**
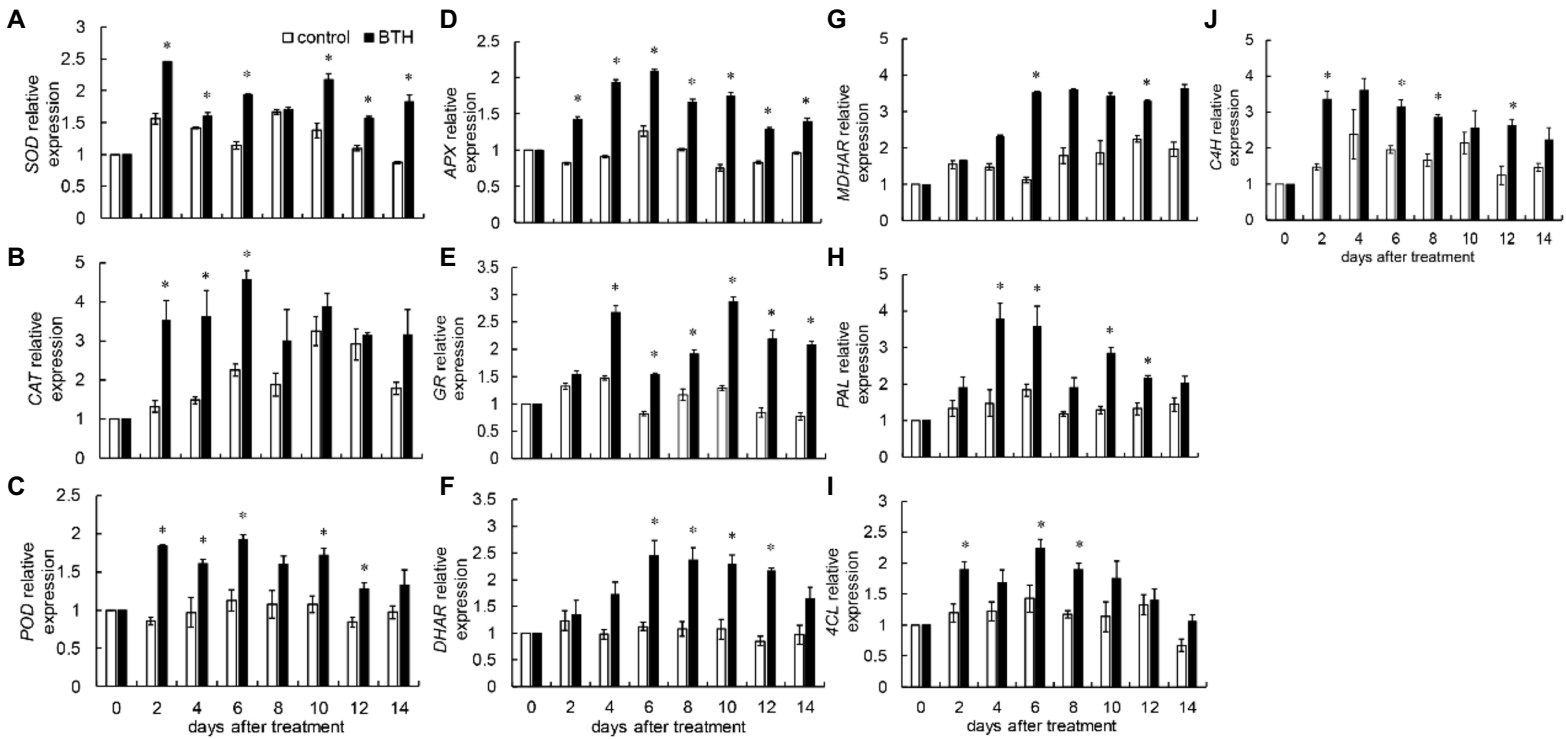
Effects of BTH treatment on the expression of *RrSOD* **(A)**, *RrCAT* **(B)**, *RrPOD* **(C)**, *RrAPX* **(D)**, *RrGR* **(E)**, *RrDHAR* **(F)**, *RrMDHAR* **(G)**, *RrPAL* **(H)**, *Rr4CL* **(I)**, and *RrC4H* **(J)** in *R. roxburghii* fruit during low temperature storage. The significant differences at a level of 0.05 by LSD between the control and BTH-treated fruit are indicated by asterisks (*). Vertical bars represent the standard errors of the means (±SE).

Benzothiazole treatment increased *RrSOD* expression. The BTH-treated fruit reached their maximum value on day 2, which was 1.6 times that of the control fruit. *RrSOD* expression was greater than that in the control fruit on days 2–6 and 10–14 (Figure 5A). After BTH treatment, *RrCAT* expression increased rapidly during the first 6days and reached its peak on day 6, which was two times as much as that of the control fruit. BTH largely increased *RrCAT* expression on days 2–6 (Figure 5B). Compared with the control fruit, BTH considerably enhanced *RrPOD* expression on days 2–6 and 10–12 (Figure 5C). BTH treatment considerably increased *RrAPX* expression during storage. On day 6, BTH-treated fruit exhibited the greatest relative gene expression, which was 1.6 times greater than the control fruit (Figure 5D). BTH treatment considerably increased *RrGR* expression on days 4–14. On day 10, the *RrGR* expression of the BTH-treated fruit reached its maximum value, which was 2.2 times that of the control fruit (Figure 5E). The *RrDHAR* expression of the treated fruit increased on days 0–6 and then decreased on days 6–12. The gene expression of the BTH-treated fruit was substantially higher than that of the control fruit. During preservation, the expression of *RrDHAR* in the control fruit did not change considerably (Figure 5F). Postharvest BTH treatment largely increased *RrMDHAR* expression on days 6 and 12, which were 3.1 and 1.4 times greater than that of the control fruit, respectively (Figure 5G). After BTH treatment, *RrPAL* expression in the BTH-treated fruit was considerably greater than that in the control group on days 6–8 and 10–12 (Figure 5H). Postharvest BTH treatment considerably increased *Rr4CL* expression on days 2, 6, and 8, reaching its peak on day 6, which was 1.5 times higher than that of the control (Figure 5I). BTH treatment notably increased *RrC4H* expression on days 2, 6, 8, and 12, i.e., 2.2, 1.6, 1.7, and 2.1 times greater than that of the control fruit, respectively (Figure 5J).

## Discussion

Moisture is an important guarantee for maintaining fruit freshness. Weight loss, which is mostly caused by respiration and transpiration, influences fruit quality. The current study found that BTH treatment can effectively reduce the weight loss rate of *R. roxburghii* fruit during storage. Du et al. (2021) determined that postharvest BTH treatment can reduce weight loss in oranges. Similar results have also been reported for apple fruit (Li et al., 2020b). BTH has been reported to induce systemic acquired resistance (SAR) in various plants (Lavergne et al., 2017), so we examined the effect of BTH treatment on decay rate in *R. roxburghii* fruit. Consistent with those reported in apples (Cao et al., 2011), strawberry (Romanazzi et al., 2013), muskmelon (Ren et al., 2012), and litchi (Xu et al., 2021), BTH inhibited *R. roxburghii* fruit decay. TA is primarily produced through the tricarboxylic acid cycle, and it can preserve the sugar-to-acid ratio in fruit, which is a key indicator for evaluating fruit quality (Ge et al., 2019a). The current research found that TA in the BTH-treated and control fruit declined during storage, with the BTH-treated fruit exhibiting lower TA than the control fruit. TSS is an important indicator for determining fruit maturity, and it directly affects fruit shelf quality. Similar to the findings for TA, the current research suggested that BTH treatment can significantly minimize the loss of TSS during storage. Previous studies have indicated that BTH treatment can effectively reduce TA content and increase TSS content in orange (Du et al., 2021), apple (Li et al., 2020b), and muskmelon (Li et al., 2015) fruit.

Cell membrane permeability and MDA can reflect the senescence level of fruit. MDA is a significant index of membrane lipid oxidative damage and peroxidation (Li et al., 2020b). The current study found that BTH treatment can effectively reduce the MDA content of *R. roxburghii* fruit during storage. Cell integrity is an important indicator of fruit quality. The present work determined that the cell membrane integrity of the BTH-treated and control fruit was decreased with prolonged storage. However, the cell membrane integrity of the BTH-treated fruit was significantly greater than that of the control fruit. Li et al. (2020a) demonstrated that treating pear fruit with BTH can essentially reduce MDA level during storage. The preceding results indicate that BTH treatment can effectively delay the senescence and maintain the quality of *R. roxburghii* fruit.

Plant cells can accumulate large amounts of ROS as a result of biological and abiotic stressors, as asserted by Li et al. (2019). Low levels of ROS act as signal molecules from the following three aspects: (1) Activating ROS sensors to induce signal cascades and affect gene expression; (2) The components of signaling pathways could be directly oxidized by ROS; and (3) ROS modulates gene expression by regulating the activity of transcription factors (Apel and Hirt, 2004); thereby causing the production of defense genes and assisting in the cross-linking and lignification of cell walls, helping cells resist fungal infections (Jiang et al., 2017; Waszczak et al., 2018). However, excessive ROS can cause damage to cells (Jiang et al., 2017). The current study found that BTH treatment can rapidly increase the O_2_^•−^ production rate of *R. roxburghii* fruit on days 0–4. In addition, H_2_O_2_ content was significantly greater than that of the control fruit during the majority of the storage period after BTH treatment. Wei et al. (2019) reported that postharvest BTH treatment increased H_2_O_2_ content in apple fruit. Ge et al. (2015) found that O_2_^•−^ and H_2_O_2_ increased after the BTH dipping treatment of muskmelon fruit. Furthermore, a similar outcome was recorded in citrus fruit after the treatment of *Pichia membranaefaciens* (Luo et al., 2013).

The antioxidant defense system is composed of enzymes and non-enzymatic antioxidants that play an important role in scavenging ROS. Enzymatic reactive oxygen scavenging systems include SOD, POD, CAT, APX etc. (Apel and Hirt, 2004). SOD directly acts on O_2_^•−^ to degrade it into H_2_O_2_. Subsequently, H_2_O_2_ is catalyzed and decomposed into H_2_O and O_2_ by CAT and APX, reducing excess H_2_O_2_ in plants and eliminating the damage caused by excess H_2_O_2_ to cells (Li et al., 2020b). POD is involved in various cell processes, such as the removal of H_2_O_2_, phenol oxidation, and lignification (Radhakrishnan et al., 2011; Wei et al., 2019). Our work determined that BTH treatment can increase the activity and expression of POD. Similar results have been found in studies on tomato (Małolepsza, 2006) and muskmelon (Ren et al., 2012) fruit. In the present study, BTH treatment increased the activities and gene expression of SOD, CAT, and APX. Ge et al. (2019b) found that postharvest BTH dipping treatment increased SOD, CAT, and APX activities, extending the storage life of blueberries. Similar results have been reported in pears (Li et al., 2020a), muskmelons (Ge et al., 2015), and peaches (Gao et al., 2016).

Non-enzymatic substances containing AsA and GSH have been reported to be involved in antioxidant scavenging in plants. AsA preserve the membrane structure and GSH directly decompose H_2_O_2_ into water. These two compounds contribute significantly to eliminate ROS in cells and protect the integrity of cell membranes (Wei et al., 2019). In our study, BTH treatment boosted the AsA and GSH contents of *R. roxburghii* fruit. Previous research has linked AsA and GSH contents to senescence in apples (Wei et al., 2019), pears (Li et al., 2020a), blueberries (Ge et al., 2019b), and strawberries (Romanazzi et al., 2013). This suggested that the delay of *R. roxburghii* fruit senescence by BTH treatment may be related to the increase of AsA and GSH content. The AsA-GSH cycle enzymes APX, MDHAR, DHAR, and GR play an important role in maintaining the dynamic balance between GSH and GSH disulfide in plants (Tao et al., 2019). Ren et al. (2012), Ge et al. (2019b), and Wei et al. (2019) reported that BTH treatment increased the activity and gene expression of GR, MDHAR, and DHAR to maintain the dynamic balance of AsA and GSH of the AsA–GSH cycle in apple and muskmelon fruit. Our result also found BTH increased the activities and gene expression of MDHAR, DHAR, and GR during the storage period. The AsA-GSH cycle enzymes APX, MDHAR, DHAR, and GR are directly related to the AsA and GSH degradation and recycling and that is why, the content of AsA and GSH are related to the activity of the AsA-GSH cycle enzymes (Nahar et al., 2015). One of the mechanisms of SAR is the induction of ROS production when plants are subjected to biotic or abiotic stress (Li et al., 2020c). BTH has been reported to activate the SA-mediated SAR response in plants, and further activate the activity and gene expression related to the metabolism of ROS (Terry et al., 2004). In our study, BTH induces a moderate increase in ROS content without damaging the organism, which might serve as a signal molecule to continuously induces the production of antioxidant scavenging enzymes to maintain cellular redox homeostasis, thereby improving the defense capabilities and delaying senescence in *R. roxburghii* fruit.

The phenylpropanoid pathway is a secondary metabolic pathway in plants. Many major antibacterial substances in plants, such as lignin, flavonoids, and phenolic compounds, are directly or indirectly produced by the phenylpropanoid pathway (Vogt, 2010). PAL is a rate-limiting enzyme in the phenylpropanoid pathway. C4H is the second key enzyme in the phenylpropanoid pathway, and its substrate is trans-cinnamic acid. 4CL can catalyze various hydroxycinnamic acids to generate the corresponding thioesters, entering the synthesis pathways of phenols, flavonoids, and anthocyanins (Liu et al., 2014). The present research demonstrated that BTH treatment can increase PAL and 4CL activities and gene expression in *R. roxburghii* fruit. Liu et al. (2014) found that postharvest BTH treatment increased the activities of PAL, C4H, and 4CL in muskmelon fruit. A similar result was obtained in the BTH treatment of blueberries (Ge et al., 2019b). Phenolic can enhance the buildup of lignin in the cell wall by acting as precursors for the synthesis of lignin and other anti-disease compounds and exhibiting a certain antibacterial effect. In addition, phenolic compounds can be oxidized into more toxic quinones under the action of polyphenol oxidase, effectively inhibiting the spread of pathogens (Liu et al., 2014). Flavonoids have antibacterial properties and can prevent spore germination, germ tubes elongation, and mycelial growth (Deng et al., 2008). In accordance with the results of the current study, the concentrations of total phenol, flavonoid, and lignin in *R. roxburghii* fruit can be increased by BTH treatment. The BTH treatment on apple (Ge et al., 2019a) and muskmelon (Liu et al., 2014) fruit exhibits similar effects.

The above results indicate that BTH treatment may delay fruit senescence by increasing the expression of key genes in the secondary metabolic pathway of phenylalanine synthesis, increasing PAL, C4H, and 4CL enzyme activity, and further promoting the content of secondary metabolites such as lignin, total phenols, and total flavonoids.

## Conclusion

**Figure 6:**
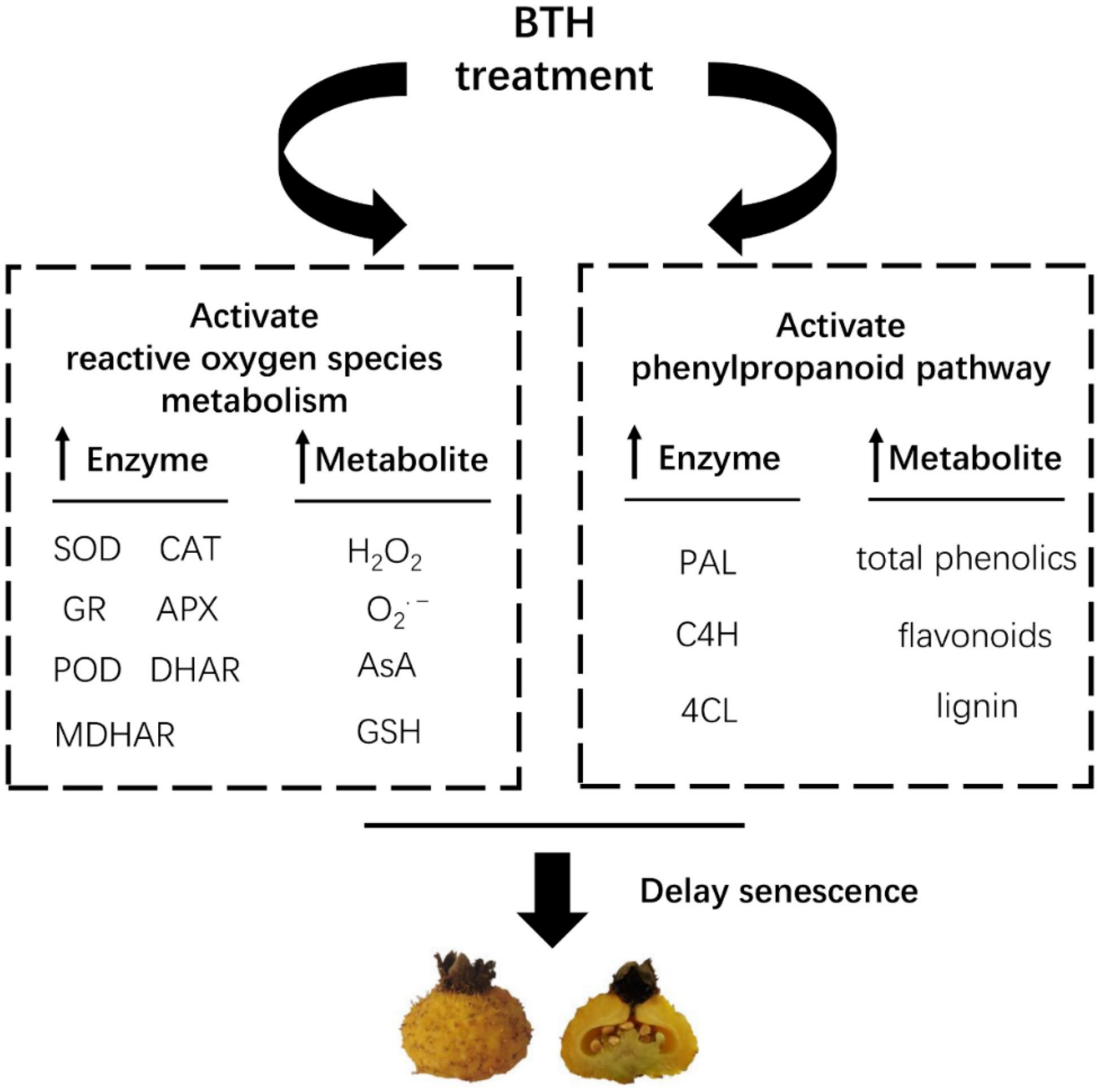
A speculation to explain the role of BTH in regulating fruit senescence in *R. roxburghii*. Arrow up indicates promotion.

Benzothiazole treatment significantly increased the activities and expression of SOD, CAT, APX, GR, POD, MDHAR, and DHAR and the contents of GSH and AsA in *R. roxburghii* fruit. It also increased O_2_^•−^ production rate and H_2_O_2_ content, but reduced MDA content. BTH treatment effectively maintained the dynamic balance of ROS metabolism and delayed the senescence of *R. roxburghii* fruit. In addition, BTH also increased the activities and expression of PAL, C4H, and 4CL and the contents of total phenols, flavonoids, and lignin, enhancing the disease resistance of *R. roxburghii* fruit (Figure 6). Nevertheless, the investigation of the mechanism of BTH to delay *R. roxburghii* fruit senescence is still needed to be further studied at the molecular level. Future studies will consider using high-throughput sequencing technologies such as metabolome sequencing and RNA-seq to identify key gene regulatory networks and metabolic regulatory networks, revealing the molecular mechanisms by which BTH treatment delays senescence in *R. roxburghii* fruit.

## Data Availability Statement

The original contributions presented in the study are included in the article/Supplementary Material; further inquiries can be directed to the corresponding authors.

## Author Contributions

BD: conceptualization, funding acquisition, resources, and writing – original draft. XD: investigation, data curation, formal analysis, methodology, funding acquisition, and writing – review and editing. HT: software, investigation, and methodology. DZ: resources and validation. QY: software and methodology. HH: methodology and validation. KH: supervision and resources. All authors contributed to the article and approved the submitted version.

## Funding

This study was supported by the Science and Technology Program of Guizhou Province, China (2021172); National Natural Science Foundation of China (32002103), and Guangdong Basic and Applied Basic Research Foundation (2020A1515110092); and Major Research Project of Innovation Group of Guizhou Provincial Department of Education (2018017).

## Conflict of Interest

The authors declare that the research was conducted in the absence of any commercial or financial relationships that could be construed as a potential conflict of interest.

## Publisher’s Note

All claims expressed in this article are solely those of the authors and do not necessarily represent those of their affiliated organizations, or those of the publisher, the editors and the reviewers. Any product that may be evaluated in this article, or claim that may be made by its manufacturer, is not guaranteed or endorsed by the publisher.
