# Supplementary material for: Benzothiazole Treatment Regulates the Reactive Oxygen Species Metabolism and Phenylpropanoid Pathway of Rosa roxburghii Fruit to Delay Senescence During Low Temperature Storage
Source: Front Plant Sci. 2021 Oct 25;12:753261. doi: 10.3389/fpls.2021.753261 (PMC8573082; doi:10.3389/fpls.2021.753261)
Supplement: Supplementary file 1 [file Data_Sheet_1.docx]

Supplementary Material

# Supplementary Data

Supplementary Data are deposited to FigShare for permanent storage and DOI is <https://figshare.com/s/8c505eca7639be54f3a7>.

**Supplementary Table 1.** Primer sequences used for real-time PCR

| Gene | Primer sequence | GenBank ID |
| --- | --- | --- |
| *RrSOD* | Forward: GGATCTTATCGGGCGGTCAATAGC | XM_009375054 |
|  | Reverse: TCCAGCACTTCTAGCAATCACAGC |  |
| *RrCAT* | Forward: GTGCTGACTTCCTTCGAGCC | KM670006 |
|  | Reverse: TTCACTGCAAAACCACGAGGA |  |
| *RrAPX* | Forward: CTGGCAAGGGTTCTGACCAC | GU552462 |
|  | Reverse: CATCCTTGTGTGCCCTTCCC |  |
| *RrGR* | Forward: TCACTCGGTCTCTTCCATCCCTTC | XM_009376342 |
|  | Reverse: GACTGGTGTGGCTTGGCTTCTTC |  |
| *RrMDHAR* | Forward: GGCTGTGATTGTTGGAGGAGGATAC | GU552461 |
|  | Reverse:TCATAGAAGGCAGCAATACCAGATGTG |  |
| *RrDHAR* | Forward: GTACCCTACAAGCTCCACCTCATAAAC | GU552460 |
|  | Reverse: AGCAAGCACATCAGAATCAGAGACC |  |
| *RrPOD* | Forward: AACTTCAACGGCACAGGAAACCC | KC153029 |
|  | Reverse: TGCTGAGATCGAGGTTGGCTAGAG |  |
| *RrPAL* | Forward: AGCAGCACAACCAGGATGTCAAC | KJ939348 |
|  | Reverse: TCCTCCAAATGCCTCAAGTCAATAGC |  |
| *RrC4H* | Forward: ACTGGTGAATGCTTGGTGGTTGG | KF663548 |
|  | Reverse: GATACCTGAAGTCGTTCCCGTTAGC |  |
| *Rr4CL* | Forward: TCCACTCCTACTGCCTCCACAAC | XM_009372686 |
|  | Reverse: GGTAAGTTCCACATCGGCGAAGG |  |
| *Rractin* | Forward: TGGTATTGTGCTTGACTCTGGTGATG | GU552463 |
|  | Reverse: CAAGACGAAGGATGGCATGAGGAAG |  |


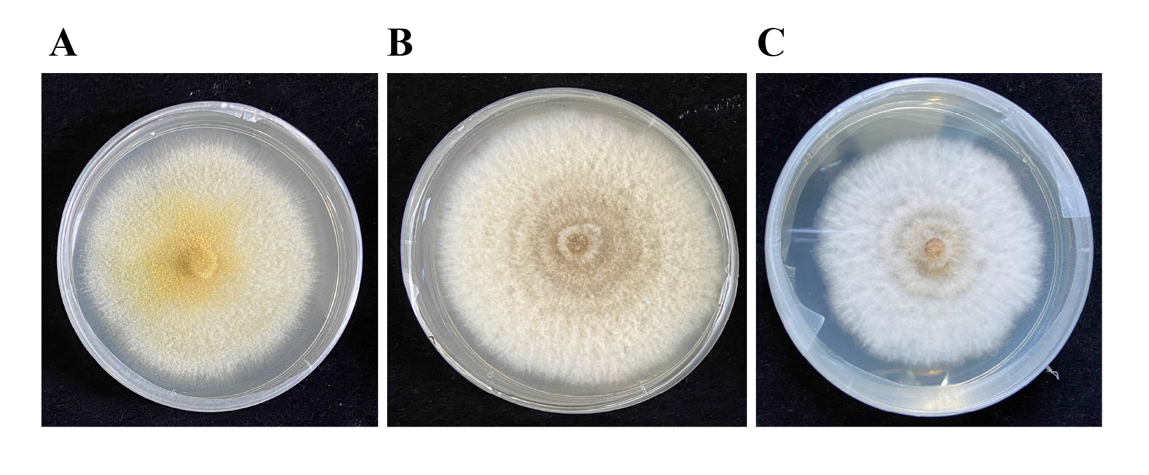


**Supplementary Figure 1.** Isolated and identified of the main microorganisms from *Rosa roxburghii* fruit. A, *Aspergillus flavus*; B, *Pestalotiopsis kenyana*; C, *Pestalotiopsis telopeae*.
